# An imaging and genetic-based deep learning network for Alzheimer's disease diagnosis

**DOI:** 10.3389/fnagi.2025.1532470

**Published:** 2025-03-21

**Authors:** Yuhan Li, Donghao Niu, Keying Qi, Dong Liang, Xiaojing Long

**Affiliations:** ^1^Research Centers for Medical AI, Shenzhen Institute of Advanced Technology, Chinese Academy of Sciences, Shenzhen, China; ^2^The Key Laboratory of Biomedical Imaging Science and System, Chinese Academy of Sciences, Shenzhen, China

**Keywords:** multi-scale deep convolutional networks, Alzheimer's disease, MRI, SNP, transformer

## Abstract

Conventional computer-aided diagnostic techniques for Alzheimer's disease (AD) predominantly rely on magnetic resonance imaging (MRI) in isolation. Genetic imaging methods, by establishing the link between genes and brain structures in disease progression, facilitate early prediction of AD development. While deep learning methods based on MRI have demonstrated promising results for early AD diagnosis, the limited dataset size has led most AD studies to lean on statistical approaches within the realm of imaging genetics. Existing deep-learning approaches typically utilize pre-defined regions of interest and risk variants from known susceptibility genes, employing relatively straightforward feature fusion methods that fail to fully capture the relationship between images and genes. To address these limitations, we proposed a multi-modal deep learning classification network based on MRI and single nucleotide polymorphism (SNP) data for AD diagnosis and mild cognitive impairment (MCI) progression prediction. Our model leveraged a convolutional neural network (CNN) to extract whole-brain structural features, a Transformer network to capture genetic features, and employed a cross-transformer-based network for comprehensive feature fusion. Furthermore, we incorporated an attention-map-based interpretability method to analyze and elucidate the structural and risk variants associated with AD and their interrelationships. The proposed model was trained and evaluated using 1,541 subjects from the ADNI database. Experimental results underscored the superior performance of our model in effectively integrating and leveraging information from both modalities, thus enhancing the accuracy of AD diagnosis and prediction.

## 1 Introduction

Alzheimer's disease (AD) is a prevalent progressive degenerative condition of the central nervous system, constituting ~60%–80% of all dementia cases (Gopalakrishna et al., 2022). As the elderly population continues to grow, the likelihood of developing this disease among older individuals is steadily increasing. Characterized by a protracted and irreversible course, AD presents limited treatment options with varying degrees of efficacy (Burns, 2020). Mild Cognitive Impairment (MCI) is often viewed as an intermediate phase between normal aging and AD, further categorized into progressive MCI (pMCI) and stable MCI (sMCI) based on the likelihood of progression to AD. Early interventions in the initial stages of AD are widely recognized as most effective, underscoring the crucial clinical significance of predicting MCI conversion (Better, 2024). Magnetic Resonance Imaging (MRI) serves as a widely adopted non-invasive imaging modality capable of identifying structural changes such as cortical thinning, brain atrophy, and regional tissue density alterations resulting from neurodegenerative diseases (Frizzell et al., 2022). With the evolution of deep learning technology, numerous studies have used deep learning networks for AD diagnosis and prediction (Wen et al., 2020; Zhang et al., 2021; Lian et al., 2020; Zhu et al., 2021). Owing to the limited number of pMCI and sMCI samples, several investigations have employed networks with architecture akin to those utilized for AD differentiation (Lian et al., 2020; Aderghal et al., 2018). In these instances, models were initially pre-trained on AD and normal samples, then subsequently applied to pMCI and sMCI classification tasks through transfer learning techniques, thereby integrating AD diagnosis and MCI conversion prediction within a unified.

To enable the earlier identification of AD and its associated risk factors, it is imperative to uncover new biomarkers at the micro level (Gatz et al., 2006). Identifying susceptibility genes and their risk variants linked to AD can aid in predicting the likelihood of developing AD before significant structural or functional changes manifest in the brain. Previous research has indicated that 60%–80% of the risk of developing AD is genetically influenced, with several genes such as APOE, APOC1, and CLU identified as being associated with AD (Zhou X. et al., 2023). Single Nucleotide Polymorphism (SNP) denotes DNA sequence polymorphisms arising from variations in a single nucleotide at the genomic level. When an SNP occurs within or in proximity to a gene's regulatory region, it may impact gene expression levels and be linked to the genetic mechanisms of the disease. The utilization of Genome-wide association study (GWAS) techniques has facilitated the identification of AD-related SNPs by comparing groups of individuals with dementia against those who are cognitively unimpaired. However, GWAS does not account for epistatic interactions. Multiple regression methodologies have been developed, integrating the apolipoprotein E (APOE) ε4 haplotype—a recognized significant sporadic AD risk factor—alongside various other AD risk SNPs identified through GWAS and polygenic risk scores. These approaches aim to provide a more comprehensive understanding of heritability and the genetic structure of AD (Yamazaki et al., 2019). Nevertheless, these methods only capture a portion of disease heritability, signifying that additional risk SNPs and critical data on interaction effects remain undiscovered.

Recently, numerous studies have integrated brain imaging and genomics data to develop deep learning methods for disease prediction and diagnosis. Ning et al. (2018) employed the volumes of 16 regions of interest (ROIs) obtained from MRI and known pathogenic SNPs as inputs to a multilayer perceptron (MLP) for AD classification. Addressing heterogeneity between different modalities, Zhou et al. (2019) proposed a three-stage deep feature extraction and fusion framework. Venugopalan et al. (2021) utilized imaging, SNP, and electronic health record data to construct deep feature extraction networks based on CNN and denoising autoencoders, subsequently classifying the features using methods such as random forests and support vector machines. Ying et al. (2021) leveraged a pre-trained 2D-CNN network to extract MRI features and an MLP for SNP feature extraction, integrating the classification results of the two models through a gating mechanism. Li et al. (2021) introduced a transformer-based SNP feature extraction network and an MRI feature extraction network based on the soft-thresholding algorithm, followed by feature fusion utilizing deep learning networks. Additionally, Zhou R. et al. (2023) proposed the ADCCA model for AD diagnosis, incorporating MRI, PET, and SNP data. This network combined MLP and canonical correlation analysis (CCA), integrating an attention mechanism and utilizing 90 ROIs extracted from MRI and 54 SNP on the APOE gene. The integral gradient method was employed to identify crucial ROIs and SNP within the classification network.

However, there are limitations within current research. Firstly, prevailing methods typically utilized ROI-based artificially set or extracted features as network inputs, such as volume and gray matter intensity. Nevertheless, the brain presents a complex network with intricate connections, and disease-related structural changes may be dispersed across different areas of the brain. Consequently, this approach may not comprehensively extract all morphological abnormalities associated with AD from the images due to patient heterogeneity. Secondly, SNP data exhibits high dimensionality but relatively small sample sizes. As a result, numerous studies have employed methods based on prior knowledge to reduce the dimensionality of SNP data, such as selecting AD-related variants listed in the AlzGene database (http://www.alzgene.org/). Furthermore, most existing methods have utilized concatenation to fuse features of the two modalities, imaging and genetics, without fully leveraging the intrinsic connection between them. This limitation consequently leads to a relatively constrained classification performance.

The primary contributions of this work can be summarized as follows:

1. Introduction of a multi-modal deep learning network aimed at enhancing the performance of AD diagnosis and prediction. This network was designed to capture more comprehensive brain structure and genetic information from whole-brain MRI and SNP data.
2. Development of a transformer-based fusion module tailored for integrating genetic data and structural images to extract intrinsic information between MRI and SNP.
3. Utilization of an interpretability approach based on grad-cam and attention map to explore and present brain structures and risk variants potentially associated with the disease.

## 2 Materials and methods

### 2.1 Overall architecture

**Figure 1:**
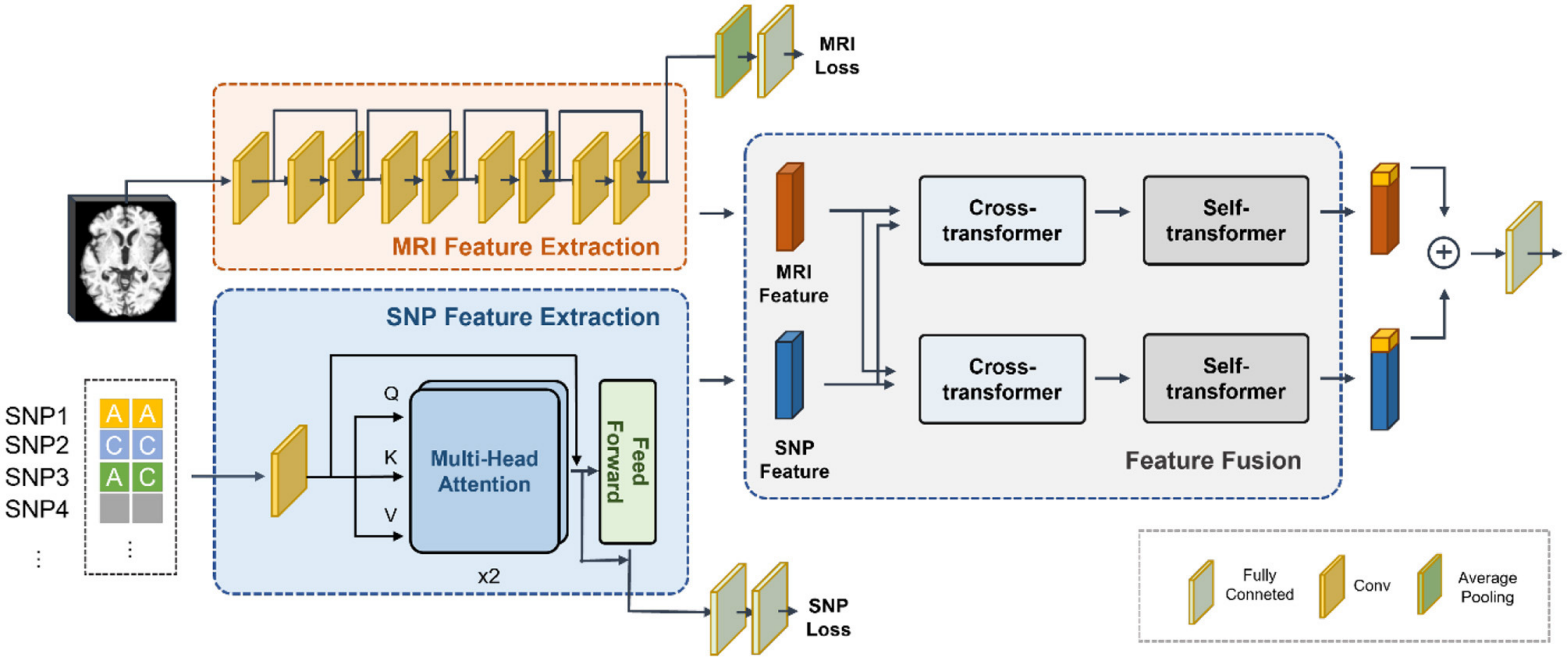
Overview of our proposed multi-modal deep learning network based on MRI and SNP. We first trained a convolutional neural network to extract whole-brain structural features and a Transformer network to extract genetic features. Then we employed a cross-transformer-based network for feature fusion. Finally, the CLS tokens from the two features were taken and concatenated to obtain the classification results.

The fundamental structure of the proposed network was depicted in Figure 1, comprising three main modules: MRI feature extraction, SNP feature extraction, and feature fusion. Preprocessed whole-brain MRI and SNP data were utilized as inputs. To extract MRI features, we employed ResNet as the backbone, while a Transformer network was used for SNP feature extraction. Furthermore, our approach involved a cross-transformer-based network for feature fusion, drawing inspiration from LXMERT (Tan and Bansal, 2019).

Due to the scarcity of samples containing genetic data and the substantial number of variants compared to the sample size, the SNP feature extraction module was susceptible to overfitting. Simultaneously, training the MRI feature extraction module necessitated a significant volume of data. Considering the imbalance in the parameters of the two networks, we initially trained the feature extraction networks for the two modalities independently. Subsequently, we determined the parameters of the feature extraction module and proceeded to train the feature fusion module.

### 2.2 Subjects and pre-processing

Data used in this study were sourced from the Alzheimer's Disease Neuroimaging Initiative (ADNI) database (Jack et al., 2008; Weiner et al., 2017), encompassing T1-weighted structural MR scans and SNP data obtained from 1636 subjects at their baseline acquisitions spanning three ADNI phases (i.e., ADNI-1, ADNI-2, and ADNI-3). Subjects were categorized into four groups—NC, AD, pMCI, and sMCI—based on standard clinical criteria, including Mini-Mental State Examination (MMSE) scores and Clinical Dementia Rating (CDR) scores. Normal controls are defined by MMSE scores of 24–30, a CDR of 0, and absence of depression or dementia. Individuals with MCI exhibit MMSE scores of 24–30, a CDR of 0.5, subjective memory complaints, and objective memory deficits confirmed by standardized assessments. Within the MCI cohort, sMCI refers to individuals who retained their MCI status during follow-up clinical evaluations, whereas pMCI denotes those who transitioned to AD at subsequent time points. AD is diagnosed based on MMSE scores ≤ 26, CDR >0.5, and fulfillment of the National Institute of Neurological and Communicative Disorders and Stroke–Alzheimer's Disease and Related Disorders Association (NINCDS/ADRDA) criteria for probable AD, including progressive cognitive decline that interferes with daily functioning. In the end, we got 610 NC subjects, 239 AD patients, 298 pMCI participants, and 489 sMCI participants. Demographic details of the subjects were presented in Table 1.

**Table 1:** Demographic information of the dataset.

**Group**	**#of subj**	**Gender (male/female)**	**Age (mean ±SD)**	**MMSE (mean ±SD)**	**CDR (mean ±SD)**
NC	610	284/326	75.8 ± 5.0	29.10 ± 1.01	0.02 ± 0.00
AD	239	125/114	75.3 ± 7.5	23.28 ± 2.03	0.75 ± 0.25
pMCI	298	179/119	74.8 ± 6.8	26.59 ± 1.71	0.50 ± 0.00
sMCI	498	278/211	74.9 ± 7.6	27.27 ± 1.78	0.49 ± 0.04

We adhered to the standard MRI pre-processing pipeline, commencing with MRI intensity correction using the N3 algorithm (Sled et al., 1998). Subsequently, skull-stripping was conducted utilizing the ANTs software (http://stnava.github.io/ANTs/), followed by linear registration to the Colin27 template (Holmes et al., 1998) through FLIRT (Jenkinson et al., 2002) in the FSL package (Jenkinson et al., 2012) to mitigate global linear disparities. Finally, we uniformly cropped the pre-processed images to eliminate extraneous background along the image edges, resulting in a standardized image size of 152 × 184 × 152 and a spatial resolution of 1 × 1 × 1 mm^3^.

For the SNP data, quality control procedures were applied to each SNP dataset using Plink software (Chang et al., 2015). These measures included: (1) Exclusion of SNPs with a missing rate exceeding 5% and samples with a genotyping detection rate below 95%, (2) Elimination of samples exhibiting gender differences, (3) Removal of SNPs with a *p*-value < 1e-6 in the Hardy–Weinberg equilibrium test, (4) Exclusion of SNPs with a minor allele frequency below 0.05, and (5) Addressing population stratification.

SNP variants for patients were not standardized across different phases of ADNI due to the limitations of microarrays for large-scale genotyping. These were sourced from Illumina Human 610-Quad, Illumina Human Omni Express, and Illumina Omni 2.5 M arrays, respectively (Saykin et al., 2010). Consequently, prior to merging, genotype imputation was conducted separately on each SNP dataset. Before imputation, SNP variants obtained from different platforms were harmonized to the GRCh37 version using Bcftools (Danecek et al., 2021), with concurrent correction of DNA strands. The Sanger Imputation Server (https://www.sanger.ac.uk/) was utilized to estimate missing genotypes, while SHAPEIT (Delaneau et al., 2012) facilitated pre-phasing during the genotype imputation process. The reference panel selected for imputation was the 1000 Genomes Phase 3 data. Subsequent to obtaining the imputed data, SNPs meeting criteria of INFO score >0.5 and genotype posterior probability < 0.9 were retained, whereas SNPs featuring more than two alleles were excluded.

Following genotype imputation, SNP data from four datasets were consolidated. Subsequently, the merged data underwent additional quality control, involving the exclusion of sites with a genotype call rate below 90%, minor allele frequency < 5%, and Hardy–Weinberg equilibrium test *p*-value lower than 1e-6. Ultimately, 4,967,369 SNP variants successfully passed the quality control process. Upon completion of preprocessing, a total of 1,541 subjects were retained, comprising 567 NC, 239 AD subjects, 293 pMCI, and 455 sMCI.

### 2.3 MRI features extraction

We employed a ResNet-based module to extract features from MRI data. Initially, a 3 × 3 × 3 convolutional kernel with 64 channels was utilized for feature extraction, followed by a batch normalization layer and a ReLU activation function. Subsequently, four residual connection modules were employed to further enhance feature extraction. This process resulted in the generation of an MRI feature map sized at 512 × 10 × 6 × 5.

During the pre-training phase of the feature extraction network, the feature maps obtained via the residual connection modules were first directed to the global average pooling layer. These feature maps were subsequently flattened to serve as input for the ensuing fully connected layers. The final layer produced two scores, which were normalized using the softmax function, representing the probabilities of negativity and positivity.

### 2.4 SNP feature extraction

Considering the significant impact of prior knowledge on SNP selection based on known susceptibility genes, there is a risk of overlooking the discovery of new risk variants. To address this, we opted to employ GWAS for SNP dimensionality reduction. SNPs that exhibited a stronger correlation with the sample phenotype based on *p*-values were filtered out.

Following this, the SNP genotype sequences were encoded using the one-hot encoding method. Each SNP was represented as a 1 × 4 vector, with the reference allele homozygote being encoded as 1,000, the heterozygote as 0100, the alternate allele homozygote as 0010, and any missing genotype as 0001. Post-encoding, the SNP sequence size for each sample became *n* × 4, where *n* denoted the number of SNPs input into the network for each subject.

After dimensionality reduction and encoding, we used a transformer network for SNP feature extraction. To enhance the nonlinearity of the network, we first used a convolutional kernel with a size of 3 and padding of 1 to transform the input data size into *n* × 32. Subsequently, two multi-head Attention block were used for feature extraction. Each block consisted of two attention heads. In the attention head structure, the input data was first mapped to three different matrices (key, query, and value) through three separate linear layers. We used dot-product and softmax function to calculate attention maps from the query and key matrices. Then the attention maps were multiplied with the value matrices. The attention-weighted features concatenated with origin features were projected to feed-forward layer. The feed-forward layer consisted of two linear layers (64 units and 32 units respectively) and a residual shortcut connection with layer normalization. For input, the transformer output can be formulated as


(1) $$\begin{array}{c} y=LaynerNorm\left(x+MSA\left(x\right)\right), \end{array}$$

(2) $$\begin{array}{c} f=LaynerNorm\left(y+Linear\left(x\right)\right). \end{array}$$

Finally, the encoded SNP features with the size of 925 × 32 were flattened into a one-dimensional vector. After passing through two linear layers and a softmax function, a score was obtained for classification.

### 2.5 Multimodal feature fusion and classification

In order to effectively integrate MRI features and SNP features, we developed a cross-transformer-based network for feature fusion. This module utilized the SNP features encoded by the transformer and the MRI features before average pooling as inputs. To ensure alignment of feature dimensions between the two modalities, we initially reshaped the MRI features to 512 × 300, while the shape of SNP features was adjusted to 925 × 32. Following typical transformer-based network procedures, we included a CLS token at the beginning of the features from both modalities, serving as a representative semantic feature for the final classification.

Two cross-transformer blocks were employed, each simultaneously taking the MRI features and SNP features as inputs. One block used the MRI features as input for the key and value matrices, and the SNP features as input for the query, while the other block used the SNP features as input for the key and value matrices, and the MRI features as input for the query. Each block consisted of four attention heads and a feed-forward layer. The processing can be expressed as


(3) $$\begin{array}{c} Q_{1}=f_{\text{snp}}W_{1},K_{1}=f_{mri}W_{2},V_{1}=f_{mri}W_{3}, \end{array}$$

(4) $$\begin{array}{c} f_{\text{snp}\to \text{mri}}=\text{Transformer}\left(Q_{1},K_{1},V_{1}\right), \end{array}$$

(5) $$\begin{array}{c} Q_{2}=f_{mri}W_{4},K_{2}=f_{\text{snp}}W_{5},V_{2}=f_{\text{snp}}W_{6}, \end{array}$$

(6) $$\begin{array}{c} f_{mri\to snp}=\text{Transformer}\left(Q_{2},K_{2},V_{2}\right). \end{array}$$

The self-transformer block used the previously obtained MRI and SNP features as inputs for further extraction of intra-modality features. It also included four attention heads and a feed-forward layer.

Following the extraction of inter-modality and intra-modality features via the cross-transformer and self-transformer, respectively, the CLS tokens from the two feature maps were extracted and subsequently concatenated. The concatenated features were then processed through a fully connected layer and softmax function to derive the final classification result.

## 3 Results

### 3.1 Implementation

The dataset, comprising both SNP and MRI data, was randomly divided into training (60%), validation (20%), and test (20%) sets. Both the pre-training and joint training stages employed cross-entropy loss as the network's loss function.

The proposed method was trained in practice using the Adam optimizer with a batch size of 4. An initial learning rate of 1*e*−4 was employed, which was subsequently reduced by a factor of 0.1 after 10 training epochs. The network implementation was carried out in Python using the PyTorch library on a single NVIDIA GTX 3090 GPU.

### 3.2 Evaluation metrics

In this study, we used four metrics to evaluate the classification performance, including accuracy (ACC), sensitivity (SEN), specificity (SPE), and F1 score. The F1 score is the harmonic average of the model sensitivity and specificity. These metrics are defined as:


(7) $$\begin{array}{cc} ACC & =\frac{TP+TN}{TP+TN+FP+FN}, \end{array}$$

(8) $$\begin{array}{cc} SEN & =\frac{TP}{TP+FN}, \end{array}$$

(9) $$\begin{array}{cc} SPE & =\frac{TN}{TN+FP}, \end{array}$$

(10) $$\begin{array}{cc} F1-Score & =\frac{2TP}{2TP+FN+FP}. \end{array}$$

where TP, TN, FP and FN denoted the true positive, true negative, false positive and false negative value respectively.

### 3.3 Competing methods

We compared the proposed method with four baseline methods, including machine learning methods and deep learning methods.

1) Machine learning methods: initially, we selected the widely used machine learning methods, including random forest (RF) and support vector machines (SVM). We employed the imaging features and genetic features obtained by the feature extraction module as inputs for both methods.

2) Concat + MLP method: following the work (Li et al., 2021), we also performed a stitching operation on MRI features and SNP features to obtain the fused features. Subsequently, we utilized an MLP network including three fully connected layers followed by a softmax function to obtain the final classification results.

3) Bilinear pooling: bilinear pooling is a commonly used feature fusion method that is primarily used to combine different feature vectors to obtain a joint representation space (Braman et al., 2021; Chen et al., 2020). On the bilinear pooling module, a gate attention mechanism was first employed to calculate the weights of MRI and SNP features, controlling the expression of features extracted from each branch. Next, the outer product was used to calculate the relationship between different modal features. Finally, the obtained fused features were projected onto two fully connected layers, and the activation function was applied to obtain the final classification results.

### 3.4 Classification performance

#### 3.4.1 AD classification

The performance of the proposed method in AD classification on the test dataset was presented in Table 2. The findings indicated that utilizing solely MRI information yielded superior classification results compared to using only SNP data. Furthermore, leveraging the combined features of both modalities leads to enhanced classification performance over using individual modality features. Specifically, our multi-modal network demonstrated an improvement in accuracy from 91.77 to 93.04% and in the F1 score from 85.71 to 88.17%, when contrasted with the utilization of imaging data alone.

**Table 2:** AD vs. NC classification performance.

**Data**	**ACC**	**SEN**	**SPE**	**F1-score**
SNP	81.01%	71.11%	84.96%	69.09%
MRI	91.77%	86.67%	93.77%	85.71%
Fusion	93.04%	91.11%	93.81%	88.17%

#### 3.4.2 MCI conversion prediction

Given that MCI serves as the preliminary stage in the development of AD, intervening prior to progression to AD can effectively mitigate the advancement of the disease. As a result, it becomes imperative to predict the likelihood of MCI evolving into AD and to differentiate between progressive MCI (pMCI) and stable MCI (sMCI). Although distinctions between pMCI and sMCI are minimal, our approach involved selecting SNP variants obtained through GWAS from the AD-NC training set. In the MRI feature extraction module, we employed transfer learning by utilizing AD-NC subjects to train the network and initialize the parameters of the pMCI-sMCI classification network.

The results for MCI conversion prediction were detailed in Table 3. Our findings suggested that the brain structural and genetic features associated with the disease, as obtained from our proposed network, were effective and hold potential for future use in predicting MCI conversion. Interestingly, we observed a decrease in accuracy when both SNP and MRI data were employed. This reduction may be attributed to the subtle distinctions in loci between pMCI and sMCI, which cannot be adequately captured using the same SNP feature extraction network utilized for AD vs. NC classification.

**Table 3:** pMCI vs. sMCI classification performance.

**Data**	**ACC**	**SEN**	**SPE**	**F1-score**
SNP	65.33%	64.41%	65.93%	59.38%
MRI	78.00%	66.10%	85.71%	70.27%
Fusion	76.67%	72.88%	79.12%	71.07%

#### 3.4.3 Comparative analysis

Compared with existing deep learning studies that primarily concentrate on MRI or SNP data, our research emphasized the fusion of features from both modalities. Table 4 provided a comparison of the results obtained by our method and other competing techniques in AD classification, revealing superior performance achieved by our network. In contrast to conventional methods such as machine learning, feature concatenation, and bilinear pooling discussed in the preceding section, our utilization of a cross-transformer network has exhibited enhanced capabilities in utilizing the relationship between genes and images to achieve improved classification performance.

**Table 4:** Comparison with other methods.

**Methods**	**ACC**	**SEN**	**SPE**	**F1-score**
RF	89.87%	86.66%	91.15%	82.93%
SVM	88.61%	73.33%	94.69%	78.51%
Concat + MLP	92.40%	86.67%	94.69%	86.67%
Bilinear	92.40%	80.00%	97.34%	85.71%
Ours	93.04%	91.11%	93.81%	88.17%

### 3.5 Interpretability analysis

While numerous methods rely on deep learning networks for AD diagnosis and prediction, a notable limitation has been the lack of clinical interpretability, hindering the delivery of dependable diagnostic evidence for clinical application. In our study, we integrated an interpretability analysis approach based on Grad-CAM (Selvaraju et al., 2017). This method facilitated automatic identification of crucial structural brain changes and risk variants linked to disease progression. Additionally, we utilized ANNOVAR (http://www.openbioinformatics.org/annovar/) to annotate risk variants and their respective genes.

**Figure 2:**
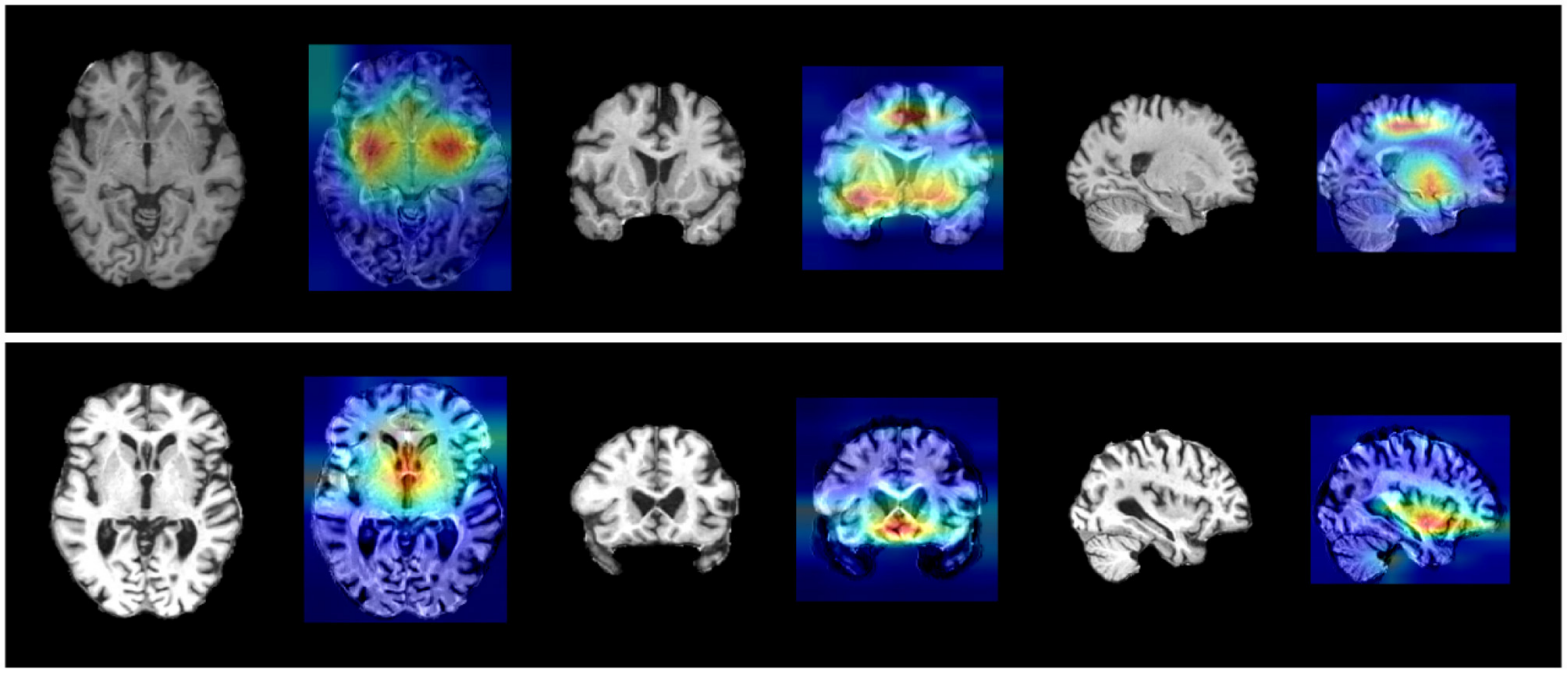
Visualization results of Grad-CAM for two groups. **(Upper)** AD vs. NC crucial brain areas. **(Lower)** pMCI vs. sMCI crucial brain areas.

The interpretability analysis outcomes were illustrated in Figure 2 and detailed in Table 5, derived by averaging data across all individual participants. Several noteworthy observations can be made based on these results. Firstly, our method successfully pinpointed key pathological brain regions, distinguishing AD patients from normal controls, as well as pMCI from sMCI, including the superior parietal cortex, basal ganglia, hippocampus, amygdala, and additional temporal areas. The detection of these regions underscored their significance as neuroimaging biomarkers in AD progression and validated the robustness of the localization results generated by our method.

**Table 5:** Key SNPs obtained using interpretability methods.

**SNP**	**Chr**	**Gene**
rs769449	19	APOE
rs59007384	19	TOMM40
rs2075650	19	TOMM40
rs566177061	6	TSBP1-AS1
rs55825602	19	TOMM40
rs6955647	2	TANC1
rs439401	19	APOE
rs73142265	6	TSBP1-AS1
rs4676754	19	KLK3
rs192303	7	NPSR1
rs157582	19	TOMM40
rs3760720	19	LOC105372441; KLK3
rs484195	19	APOC1
rs2072153	17	ZNF652
rs2980879	8	TRIB1; LINC00861
rs1264435	6	ABCF1
rs4939291	11	OR4D6; OR4D10
rs13111134	4	UGT2B4
rs113785991	3	ABCC5
rs73142265	7	TPST1

Secondly, as indicated in Table 5, the interpretability analysis also revealed crucial variant loci associated with AD. Top SNPs linked to AD included rs769449 (APOE, associated with p-tau181 levels), rs59007384 (TOMM40, CSF APOE correlation), and rs2075650 (TOMM40, AD risk allele; Table 5, Figure 2). Novel loci (e.g., rs566177061, rs113785991) were also identified.

**Figure 3:**
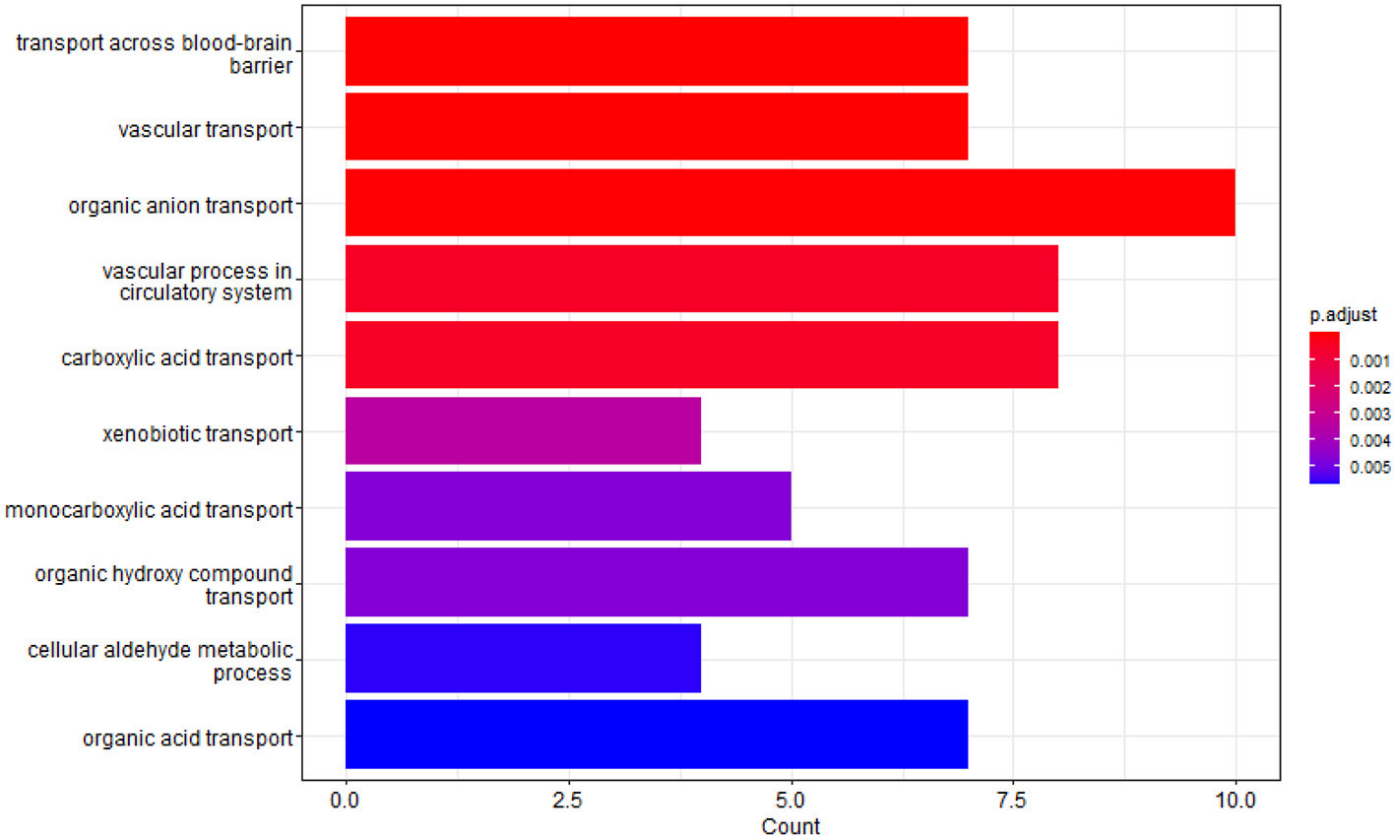
GO enrichment analysis results in biological process.

**Figure 4:**
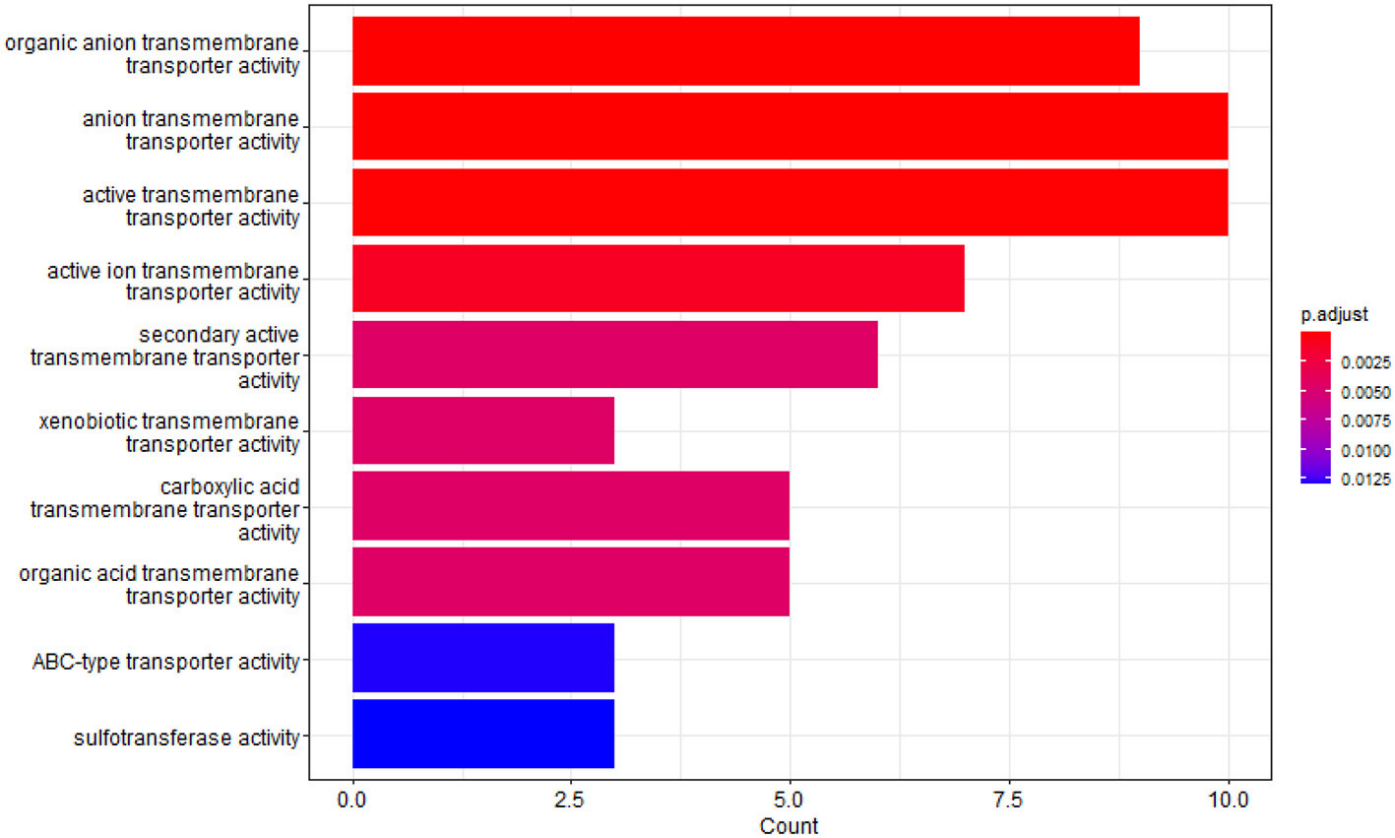
GO enrichment analysis results in molecular function.

We identified the top 100 crucial SNP sites and annotated the SNPs to the corresponding genes. After eliminating duplicate genes, a total of 75 genes remained. Subsequently, we conducted Gene Ontology (GO) enrichment analysis on these genes, setting the enrichment analysis *p*-value and FDR-corrected *p*-value thresholds at 0.05. This analysis yielded 41 biological process gene clusters and 29 molecular function gene clusters, as illustrated in Figures 3, 4, respectively. Notably, among the key genes associated with AD predicted by our network model, those related to the blood-brain barrier (BBB) transport mechanism held the highest ranking, with a corrected *p*-value of 6.98e-6. These genes include APOE (Jackson et al., 2022), ABCC5 (Zhang et al., 2023), SLC22A2 (Huttunen et al., 2022), SLC16A12 (Nguyen et al., 2022), ABCC2 (Schulz et al., 2023), SLC22A3 (Huttunen et al., 2022), and SLC16A7. In addition, our enrichment analysis also highlighted the significant roles of several mechanisms within the circulatory system, including vascular transport, organic anion transport, and vascular processes. In terms of molecular functions, the top three enriched activities were organic anion transmembrane transporter activity, anion transmembrane transporter activity, and active transmembrane transport activity.

### 3.6 Ablation experiments

#### 3.6.1 Analysis on SNP feature extraction module

To assess the efficacy of backbone models in the SNP feature extraction module, we employed MLP, TextCNN (Kim, 2014), AttentionCNN, and Transformer as individual backbones. The outcomes of the experiment were presented in Table 6. Our findings revealed that the use of the Transformer model resulted in the highest performance, attaining a maximum accuracy of 81.01% along with an F1-score of 69.09%.

**Table 6:** Comparison with other methods.

**Methods**	**ACC**	**SEN**	**SPE**	**F1-score**
MLP	74.68%	57.78%	81.41%	56.62%
TextCNN	79.75%	64.44%	85.84%	64.44%
AttentionCNN	80.34%	66.67%	85.84%	65.39%
Transformer	81.01%	71.11%	84.96%	69.09%

#### 3.6.2 Analysis on SNP dimensionality reduction

Owing to the high dimensionality of SNP data and the constrained number of subjects, the dimensionality of the SNP input significantly influenced both the efficacy of the SNP feature extraction module and the interpretability analysis results. We investigated the impact of *p*-value selection in GWAS on the AD classification performance of the SNP feature extraction module. The experimental findings were detailed in Table 7, revealing that the module exhibited optimal performance when employing a SNP set with a *p*-value lower than 1e-6, achieving a peak accuracy of 81.01% and an F1 score of 69.09%.

**Table 7:** Result of AD vs. NC classification using different SNP sets.

***p*-value**	**Number of variants**	**ACC**	**SEN**	**SPE**	**F1-score**
1e-5	1,420	79.13%	44.44%	92.92%	54.79%
1e-6	925	81.01%	71.11%	84.96%	69.09%
1e-7	593	81.01%	64.44%	87.61%	65.91%

#### 3.6.3 Analysis on the length of encoding SNP

Given our focus on SNP genotypes without specific base arrangements, we restricted the SNP genotypes to four possibilities: AA, aa, Aa, and missing. Unlike the lexical diversity in natural language models, SNP genotypes were encoded within relatively constrained parameters. To bolster the model's generalization, we introduced one-dimensional convolution at the outset of the SNP feature extraction module to extend the SNP embedding length. The selection of the number of convolutional kernel channels could influence subsequent feature extraction effectiveness. Consequently, we conducted experiments using one-dimensional convolutional kernels with varied parameters. The experimental outcomes, as detailed in Table 8, revealed that encoding SNPs with a length of 32 yielded the most effective classification performance.

**Table 8:** Result of AD vs. NC classification using different length of encoding SNP.

**Channel**	**ACC**	**SEN**	**SPE**	**F1-score**
4	79.75%	44.44%	93.81%	55.56%
16	81.01%	60.00%	89.38%	64.29%
32	81.01%	71.11%	84.96%	69.09%
64	79.75%	75.56%	81.42%	68.00%

#### 3.6.4 Analysis on feature fusion module

**Figure 5:**
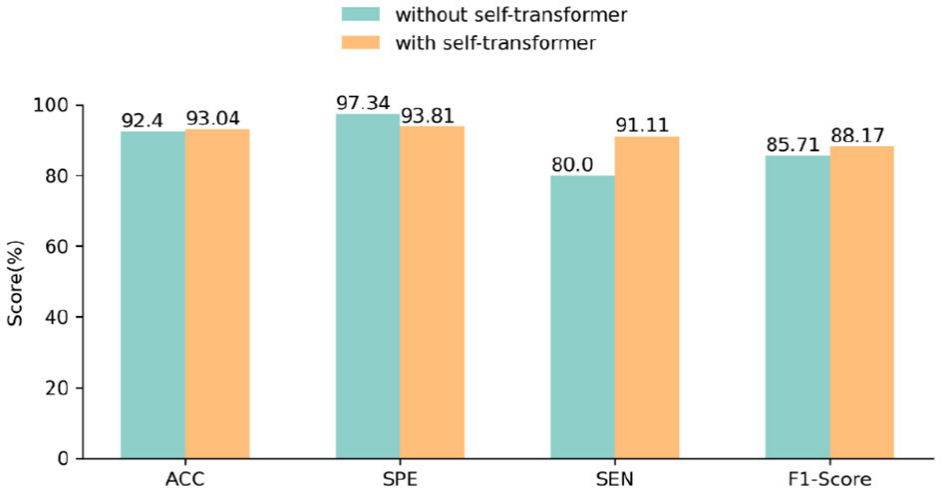
AD classification performance using networks with and without the self-transformer block.

In order to scrutinize the impact of the self-transformer block within the feature fusion module, we performed AD classification experiments employing networks both with and without the self-transformer block. As depicted in Figure 5, our experimental findings illustrated that integrating the self-transformer block enhanced the classification performance of the network, leading to an increase in accuracy from 91.77 to 93.04%. This improvement implied that the self-transformer block leveraged the fused features obtained from the cross-transformer block, thereby amplifying essential disease-relevant features through its attention mechanism.

## 4 Discussion

### 4.1 A ResNet-transformer dual-modality deep learning framework for AD classification

This study proposed a dual-modality deep learning framework integrating MRI and SNP data, which significantly improved AD classification accuracy and provides a critical technical advancement for early prediction of MCI progression to AD. The methodology combined ResNet and Transformer networks to extract high-level features from whole-brain MRI and pre-filtered SNP data, respectively, followed by cross-modality Transformer-based fusion. This approach demonstrated multiple technical innovations, revealing that deep multimodal interaction can overcome the limitations of traditional linear correlation models.

Traditional AD neuroimaging analyses predominantly rely on predefined brain measurements (e.g., volume or cortical thickness), which risk overlooking global microstructural changes and cross-regional degenerative patterns. To address this, our ResNet-based MRI feature extraction module employed residual structures to effectively capture subtle whole-brain structural variations. Experimental results showed that compared to conventional methods, e.g. voxel-based morphometry (VBM), ResNet achieves a notable improvement in sensitivity to minute gray matter density changes, substantially mitigating feature omission risks caused by prior assumptions. For SNP data, which exhibits high dimensionality, weak effects, and linkage disequilibrium, traditional genome-wide association studies (GWAS) may miss potential risk loci due to reliance on statistical threshold filtering. Our Transformer-based SNP feature extraction module dynamically evaluated global dependencies among SNP loci through self-attention mechanisms. This enabled adaptive identification of known AD-associated variants (e.g., APOE: rs769449, rs439401; TOMM40: rs59007384, rs2075650) and discovery of novel candidate polymorphisms such as rs566177061 (TSBP1-AS1) and rs6955647 (TANC1). The parallel computing architecture of Transformer enhanced efficiency in detecting SNP-SNP interaction effects compared to traditional regression models, while eliminating dependence on prior gene functional annotations (Zhou X. et al., 2023). The feature fusion module employed Cross-Transformer to model deep interactions between imaging and genetic features, complemented by Self-Transformer to strengthen intra-modality feature correlations. This dual mechanism comprehensively explored non-linear multimodal relationships, outperforming conventional concatenation or shallow fusion methods.

By synergizing these innovations, the framework not only enhances diagnostic accuracy for AD and MCI progression prediction but also provides verifiable biomarker candidates for pathological mechanism exploration.

### 4.2 Genetic insights and AD pathogenesis: bridging SNPs to molecular mechanisms

The identification of key SNPs by the proposed model is consistent with the established genetic framework of AD while revealing novel pathways. Variant on rs439401 has been closely linked to AD in previous studies, a result consistent with our findings. Furthermore, we identified previously unreported variants that may also be linked to the disease, such as rs769449, rs59007384, and rs2075650. Most of these SNPs are situated on chromosome 19, and their associated genes including APOE, TOMM40, KLK3, and APOC1 have long been recognized for their significant impact on the development and progression of AD in prior research, thus validating the relevance of the key SNPs identified by our network.

Specifically, the prominently ranked rs769449 locus has been previously associated with plasma p-tau18, with the minor allele gene A of rs769449 significantly correlated with heightened p-tau181 levels. Carriers of rs769449-A exhibited more pronounced longitudinal cognitive decline (Huang et al., 2022). TOMM40, a gene encoding a protein related to cellular vitality on the outer mitochondrial membrane, is believed to potentially lead to mitochondrial dysfunction, thus being linked to AD development. The rs59007384, positioned near the TOMM40 gene, has been found in related studies to be associated with APOE levels in cerebrospinal fluid. Research suggested that considering both the APOE gene and variations at the rs59007384 locus may offer more accurate predictions of AD risk than assessing either factor alone. In APOEε4 non-carriers, rs59007384 also elevates the risk of MCI progressing to AD (Cervantes et al., 2011). Additionally, the G allele of rs2075650, associated with reduced TOMM40 expression, may impair mitochondrial resilience to Aβ toxicity, accelerating cognitive decline (Zhou, 2021). Therefore it is also considered to increase the risk of AD (Huang et al., 2016). Furthermore, several unexplored loci, including rs566177061, rs6955647, and rs4676754, may hold potential implications for AD, as yet unaddressed in existing research.

The genes related to the BBB transport mechanism are the most relevant group of genes we identified in association with the pathology and recognition of AD. The BBB serves as a crucial interface regulating the passage of substances into and out of the central nervous system (Wu et al., 2023). Research indicated that a compromised BBB integrity leads to increased permeability, potentially facilitating the entry of harmful substances (Sweeney et al., 2018), such as Aβ, into the brain, thereby contributing to Aβ deposition and accumulation—a hallmark of AD pathology. Additionally, dysfunction in the BBB transport mechanism may impede Aβ clearance, exacerbating its accumulation in the brain (Alkhalifa et al., 2023). The novel association of ABCC5 (via rs113785991) with BBB transport mechanisms introduces a previously understudied axis in AD. ABCC5, a member of the ATP-binding cassette (ABC) transporter family, regulates Aβ efflux at the BBB (Shubbar and Penny, 2020). Our GO enrichment analysis further implicates SLC22A2 and SLC16A12 in BBB solute transport, suggesting that polymorphisms in these genes may disrupt ionic homeostasis or nutrient delivery, priming the brain for neurodegeneration. The identification of genes associated with the BBB transport mechanism holds substantial promise for early AD detection and the development of potential intervention pathways.

### 4.3 The role of key brain regions and genetic variants in AD: insights from structural MRI and SNP analysis

The medial temporal lobe (MTL), which includes the hippocampus and amygdala, and the basal ganglia are crucial in the onset and progression of AD. The hippocampus, a core component of the MTL, is pivotal for memory consolidation. Structural MRI studies consistently reveal hippocampal atrophy as one of the earliest biomarkers of AD, correlating with cognitive decline (Braak and Braak, 1991). The MTL's vulnerability stems from its high metabolic demand and dense synaptic connectivity, making it susceptible to amyloid-β (Aβ) deposition and neurofibrillary tangles (NFTs) composed of hyperphosphorylated tau. These pathologies disrupt synaptic plasticity and neuronal integrity, leading to episodic memory deficits (Jack et al., 2010). The APOE ε4 allele (rs769449, rs439401 identified by the proposed model) is strongly associated with hippocampal atrophy. APOE ε4 impairs Aβ clearance, exacerbating amyloid deposition in the MTL (Liu et al., 2013). Additionally, TOMM40 (rs2075650), located near APOE, may influence mitochondrial protein import, affecting neuronal energy metabolism and accelerating MTL degeneration (Burggren et al., 2017). The basal ganglia, traditionally linked to motor control, also participate in cognitive and limbic circuits. Structural MRI studies report basal ganglia atrophy in AD, though less pronounced than in the hippocampus. This region's involvement may reflect its connections to cortical areas governing executive function, which deteriorate as AD progresses (Vitanova et al., 2019).

### 4.4 Limitations and future work

Although the experimental results showcased promising application potential for the proposed method, it is essential to conscientiously address several limitations in future endeavors to enhance its performance further.

Initially, the selection of a backbone had a discernible impact on feature extraction, consequently influencing classification performance. In our present model, we employed ResNet as the backbone for MRI feature extraction, resulting in a relatively straightforward MRI feature extraction module and consequently limiting the classification performance of the proposed model. Currently, several multi-scale deep convolutional networks, based on whole-brain and patch approaches, have demonstrated strong performance in early AD diagnosis (Lian et al., 2020; Zhu et al., 2021). Our future efforts will focus on enhancing the MRI feature extraction module.

Furthermore, the performance of the SNP feature extraction module was constrained by the utilization of diverse gene chips across different phases in ADNI. Owing to variations in SNP variants among these genotyping chips, data consolidation from various chips can only be achieved through imputation. Moreover, numerous disease-associated risk variants may not be present in the genotyping chips. In addition to SNP data, integrating additional forms of genetic data could potentially enhance the model's performance.

Additionally, the interpretability method employed in our model exhibited a relatively coarse nature. The use of trilinear interpolation in the interpretability analysis resulted in the identification of larger brain deformation regions, posing challenges in accurately pinpointing key structures. In future endeavors, we intend to integrate patch-based methods to map interpretability analysis results to smaller regions, facilitating the detection of more nuanced structural changes associated with the disease. Regarding gene interpretability analysis, our current approach only yielded the top risk variants. Subsequently, akin to prior research, we plan to utilize several significant SNPs derived from the interpretability method of the network to conduct gene ontology enrichment and expression quantitative trait variant analyses, thereby delving deeper into the pathological mechanisms of AD.

Moreover, the utilization of diverse datasets for network training may yield varied results. Despite utilizing data from multiple stages within the ADNI dataset, the relatively small data size constrained the performance of the network. Integrating additional datasets and expanding the inclusion of in-house datasets holds the potential to augment the classification performance of the network.

## 5 Conclusion

This study introduced a novel cross-transformer-based multi-modal deep learning network, leveraging both whole-brain MRI and SNP data for Alzheimer's disease diagnosis. Diverging from conventional approaches that rely on pre-defined ROI signatures and known risk variants as network inputs, our proposed network directly incorporates whole-brain MRI data and all SNPs without prior presets. This approach mitigated the influence of prior knowledge and contributed to a more comprehensive understanding of brain regions and genetic variants associated with disease progression. Furthermore, our utilization of a larger dataset spanning multiple stages of ADNI enhanced the generalization capability of our network. The performance of the proposed method was assessed across 1541 subjects, revealing that the concurrent use of MRI and SNP data improves AD diagnosis and prediction performance compared to single-modal data usage. Additionally, we implemented an attention-based approach to enhance the interpretability of the model. The discovery of numerous new SNP variants within this network will advance our understanding of the mechanisms underlying AD.

## Data Availability

The data supporting the findings of this study are publicly available in ADNI at https://adni.loni.usc.edu/. These data were accessed under a license permission granted by the database provider, and their use complies with the terms specified in the original license.
